# The crystal structure of the selenide-based synthetic sulfosalt CuPbSb_3_Se_6_

**DOI:** 10.1107/S2056989023000361

**Published:** 2023-01-24

**Authors:** Paul Sicher, Berthold Stöger

**Affiliations:** a X-Ray Centre, TU Wien, Getreidemarkt 9, 1060 Vienna, Austria; University of Kentucky, USA

**Keywords:** crystal structure, sulfosalt, selenide, disorder

## Abstract

Single crystals of copper lead tri­anti­mony hexa­selenide, CuPbSb_3_Se_6_, were obtained as a minor phase during systematic studies of the formation conditions of selenide-based sulfosalts. The crystal structure is an unusual representative of the family of sulfosalts. Instead of the expected galena-like slabs with octa­hedral coordination, it features mono and double-capped trigonal–prismatic (Pb), square-pyramidal (Sb) and trigonal–bipyramidal (Cu) coordination. All metal positions are occupationally and/or positionally disordered.

## Abstract 2

Single crystals of copper lead tri­anti­mony hexa­selenide were obtained as a minor phase during systematic studies of the formation conditions of selenide-based sulfosalts. The crystal structure is an unusual representative of the family of sulfosalts. Instead of the expected galena-like slabs with octa­hedral coordination, it features mono and double-capped trigonal–prismatic, square-pyramidal and trigonal–bipyramidal coordination.

## 1. Chemical context

Sulfosalts (Moëlo *et al.*, 2008 ▸) are promising candidates as thermoelectric materials owing to their high electrical conductivity paired with a low thermal conductivity. Inspired by natural sulfur-based sulfosalts, we attempted to further increase the electrical conductivity by substituting Se for S. During systematic studies of the formation conditions of sulfosalts of the andorite structure type (Moëlo *et al.*, 2008 ▸), we obtained crystals of the title compound, CuPbSb_3_Se_6_, as a minor phase, by heating the precursor selenides Cu_2_Se, PbSe and Sb_2_Se_3_ in evacuated fused silica ampules. Surprisingly, the title compound does not follow the expected crystal chemistry of the structural family. In fact, crystals of the andorite family are modular structures, which are composed of galena-like slabs, with octa­hedral coordination of the metal atoms. This coordination is not observed for CuPbSb_3_Se_6_. Nevertheless, certain structural relationships can be established, as will be shown below. These structural relationships are reflected by andorite-like compounds of the Sn_3_Bi_2_Se_6_ structure type (Chen & Lee, 2010 ▸) with very similar cell parameters yet a different space-group symmetry. The structure with the closest matching cell parameters is SnPb_2_Bi_2_S_6_ (Li *et al.*, 2019 ▸) with *a* = 20.5458 (12) Å, *b* = 4.0925 (4) Å and *c* = 13.3219 (10) Å, whereby the axes have been cyclically permuted with respect to the cell of CuPbSb_3_Se_6_ presented here. SnPb_2_Bi_2_S_6_ crystallizes in a lillianite-type ^4^L (Moëlo *et al.*, 2008 ▸) structure and was investigated by the authors for its thermoelectric performance, sporting a figure of merit *ZT* of 0.3. Since CuPbSb_3_Se_6_ shows strongly disordered positions, it is possible that it exhibits similar thermoelectric properties.

It should be noted that from a structural point of view, lillianites and andorites are inter­changeable terms. However, in a mineralogical context, they define distinct sulfosalt mineral groups because the Sb that replaces Bi from the lillianite structure in andorite forms electron-pair micelles that distort the structure (Makovicky & Topa, 2014 ▸).

## 2. Structural commentary

**Figure 1:**
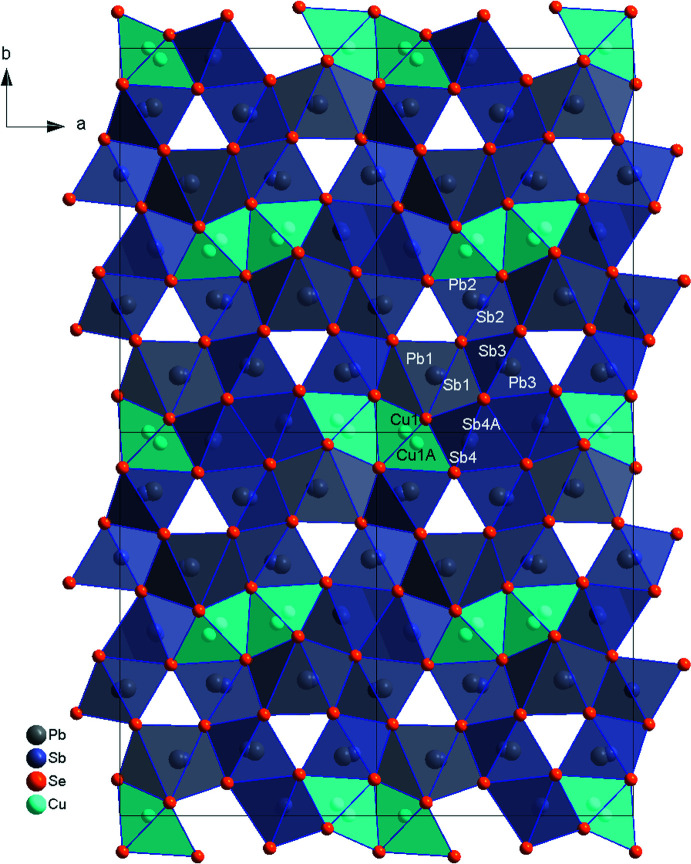
CuPbSb_3_Se_6_ viewed down [001]. Pb and Pb/Sb are represented by grey, Sb by dark blue, Cu by cyan spheres of arbitrary radius.

Crystals of the title compound crystallize in the *Pnnm* space group. All atoms are located on or disordered about (in the case of Sb4*A*) the reflection plane parallel to (001), which corresponds to the Wyckoff position 4*g*. The crystal structure is comprised of three mixed Pb/Sb positions, one Sb and one Cu position (Fig. 1 ▸). There are three different kinds of coord­ination polyhedra, with the inter­atomic distances compiled in Table 1 ▸. The predominantly Pb Pb1/Sb1 position is coordinated by Se atoms, forming a double-capped trigonal prism. The predominantly Sb Sb2/Pb2 and Sb3/Pb3 positions and the disordered Sb4/Sb4*A* are quadratic pyramids in the case of Sb and mono-capped trigonal prisms in the case of Pb. Finally, the disordered Cu1/Cu1*A* position features trigonal–bipyramidal coordination. Whereas the [PbSe_8_] double-capped trigonal prisms of the Pb1/Sb1 position are a defining feature of lillianite-type structures and form where the galena-like slabs meet, the remaining two coordinations are unexpected in this structural family.

**Figure 2:**
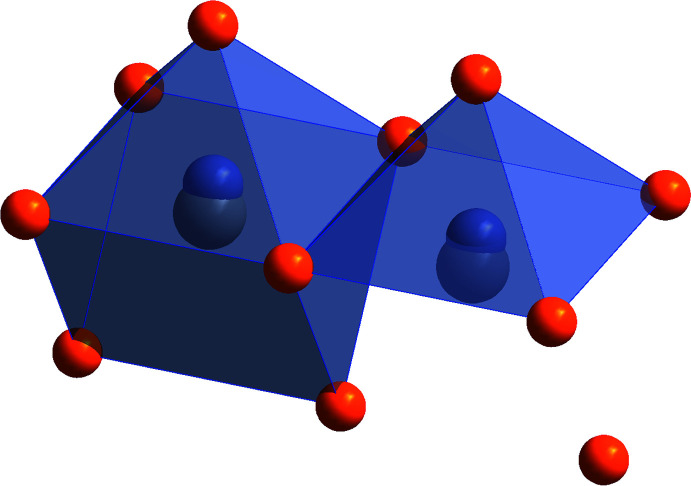
Graphical comparison of the two different coordination polyhedra of Sb3 and Pb3. Colours as in Fig. 1 ▸.

It has to be noted that the description of the coordination polyhedra of the Sb2/Pb2, Sb3/Pb3 and Sb4/Sb4*A* positions as quadratic pyramids and capped trigonal prisms is not completely unambiguous. Both variants based on the central atom are shown in Fig. 2 ▸ for Sb3 and Pb3. Since the distance from Sb3 to the two farther Se6 atoms is 3.7015 (19) Å and the corresponding calculated bond valence, using the parameters *R*_0_ = 2.60 Å and *b* = 0.37, is only 0.05, they are considered not to coordinate with Sb3. In contrast, the Se2 and Se3 atoms at the base of the pyramid are located at 2.9293 (15) Å and 2.8835 (14) Å, respectively. The Se1 atom at the apex of the pyramid is located at 2.587 (2) Å from the Sb3 atom. This is different for Pb3, where the two distant Se6 atoms are much closer, with the atomic distances changed to 3.35 (3) Å. The other Se atoms are further away with 3.05 (3) Å for Se2, 2.86 (2) Å for Se3 and 3.02 (4) Å for Se1. Note that the large standard uncertainties (s.u.s) of the Pb—Se distances here are due to Pb3 being a minor position in close proximity to Se3. Thus, in the case of the Pb3 atoms, the coordination is clearly a capped trigonal prism, whereas for Sb3 it is better described as quadratic pyramidal. When considering the electron lone-pair of the Sb^III^ atoms, the coordination might also be seen as ψ^1^-octa­hedral.

For the Sb2/Pb2 position, the same observation is made with slightly changed distances. The extended coordination environment of Sb2 possesses two far Se6 atoms at 3.6962 (17) Å, Se1 and Se4 atoms at the square base at 3.1563 (14) Å and 2.7090 (12) Å and an apex Se3 atom at 2.6135 (19) Å. For Pb2 these distances change to 3.283 (6) Å, 3.114 (7) Å, 2.900 (7) Å and 3.094 (10) Å, respectively.

On the Sb4/Sb4*A* position, the Sb atom is sometimes located on the ..*m* position [Sb4, 83 (3)%] and sometimes to both sides of the reflection plane [Sb4*A*, 2×8.4 (15)%]. The coordination of Sb4 is similar to those of Sb2 and Sb3. The coordination polyhedron can be considered as a quadratic pyramid with the bond lengths being 2×2.723 (2) Å (Se6), 2×3.166 (3) Å (Se2) and 2.595 (3) Å (Se5, located at the apex). The next Se atom is Se2 located 3.875 (2) Å from Sb4, which can be considered as non-coordinating. The coordination of Sb4*A* is very similar, as it is located only 0.44 (3) Å from Sb4.

**Figure 3:**
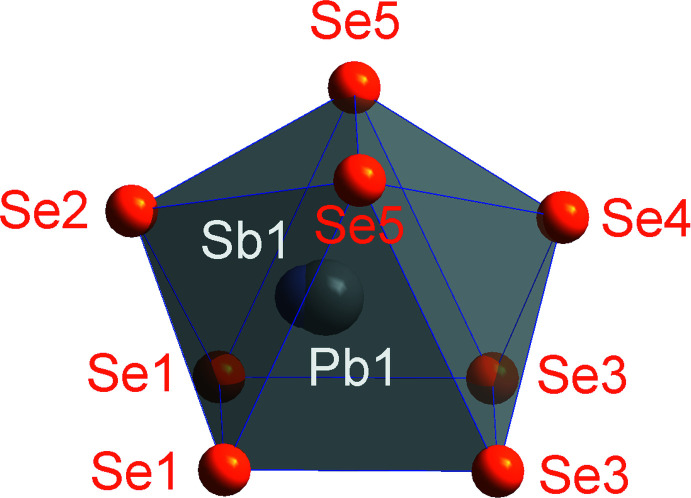
The coordination polyhedron of Pb1/Sb1. Colours as in Fig. 1 ▸

As for the other discussed coordination polyhedra, one might also see the double-capped trigonal prisms that surround the Pb1/Sb1 position (Fig. 3 ▸) as quadratic pyramids in the case of Sb because the metal atoms do not lie in the centre of the polyhedron. If the Sb1 atom is realized, one might rather think of a fivefold instead of an eightfold coordination, again with the atoms forming a quadratic pyramid. Here, the bond distances involving the Pb1 atom are 3.0553 (15) Å and 3.1205 (17) Å for the quadratic base (Se1 and Se5) and 2.9124 (14) Å to the apex (Se2). The two Se3 atoms are located at 3.5754 (15) Å from the Pb1 atom and the last Se4 atom, which forms the second cap of the prism at a distance of 3.4061 (16) Å. The coordination of Sb1 is very similar [distance to Pb1 = 0.26 (2) Å], with a slightly more pronounced quadratic pyramidal coordination.

The (double-)capped trigonal prism is, as stated above, a defining structural element of the lillianite family. It is inter­esting to note that whereas the 90° angles of all the prisms are perfectly realized owing to the ..*m* reflection plane, the triangular bases deviate significantly from an ideal trigonal symmetry. The prism around Pb1/Sb1 is formed from a triangle with 48.31 (2), 66.60 (3) and 65.09 (3)° angles. The other prisms are closer to regular, with the angles deviating the most from 60° being 54.53 (3)° for Pb2 and 65.57 (3)° for Pb3.

Finally, the trigonal–bipyramidal coordination of Cu is unusual as Cu is usually encountered as coordinated tetra­hedrally or in a planar square. This is still somewhat true for Cu1/Cu1*A*, as the disordering takes place over the trigonal base of the pyramids, placing them both in their own tetra­hedron. However, the position closer to the base (Cu1), *i.e.* with the more trigonal–bipyramidal-like coordination, has a higher occupancy [59.5 (17)%] than the position further removed from the centre of the trigonal bipyramid (Cu1*A*).

**Figure 4:**
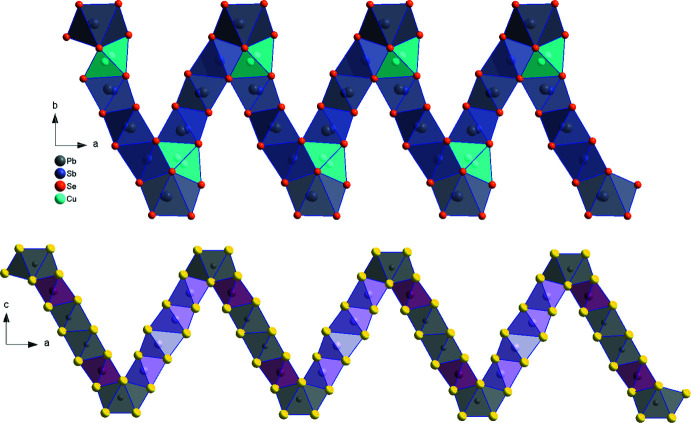
Comparison of four-polyhedra-long chains delimited by the Pb1/Sb1 position in (top) CuPbSb_3_Se_6_ and (bottom) the lillianite ^4^L-type structure of SnPb_2_Bi_2_S_6_. Colour codes: Bi red, Pb grey, Bi/Sn pink, S yellow.

Despite the clearly different coordination polyhedra, CuPbSb_3_Se_6_ can nevertheless be described as a distorted ^4^L andorite-type structure, since there are four polyhedra between two double-capped trigonal prisms as shown in Fig. 4 ▸. However, the spatial distribution of the Se/S atoms is fundamentally different, leading not only to different coordination polyhedra, as described above, but also an altered connectivity of the polyhedra.

## 3. Database survey

No compounds containing only copper, lead, anti­mony and selenium have been deposited in the Inorganic Crystal Structure Database (ICSD; Bergerhoff & Brown, 1987 ▸) as of Fall 2022.

## 4. Synthesis and crystallization

40.0mg of Cu_2_Se, 47.6mg of PbSe and 125mg of Sb_2_Se were mixed thoroughly and transferred into a fused silica ampoule, which was sealed under vacuum. The ampoule was heated at 1223 K for 2 h, cooled to 873 K over 7 h and held at that temperature for 149 h. After cooling to 473 K over 5 h and quenching in air, the ampoule was opened and the obtained ingot crushed. Among other phases in the andorite family, single crystals of the title compound CuPbSbSe_3_ were isolated.

## 5. Refinement details

Crystal data, data collection and structure refinement details are summarized in Table 2 ▸. All atoms were refined with anisotropic atomic displacement parameters (ADPs). It was necessary to model the Cu-atom position as positionally disordered to avoid non-positive definite (NPD) ADP tensors. Modelling the positions of Pb1, Sb2 and Sb3 as mixed Pb/Sb positions as well as the position of Sb4 as positionally disordered improved the residuals significantly. The pairs Pb1/Sb1, Sb2/Pb2, Sb3/Pb3 and Sb4/Sb4*A* were refined with identical ADP tensor elements, though distinct coordinates. Furthermore, the site occupancies were constrained to full occupancy and the Pb occupancies were restrained to fit the sum formula CuPbSb_3_Se_6_, corresponding to an electroneutral structure.

## Supplementary Material

Crystal structure: contains datablock(s) global, I. DOI: 10.1107/S2056989023000361/pk2677sup1.cif

Structure factors: contains datablock(s) I. DOI: 10.1107/S2056989023000361/pk2677Isup2.hkl

CCDC reference: 2236460

Additional supporting information:  crystallographic information; 3D view; checkCIF report

## Figures and Tables

**Table 1:** Selected bond lengths (Å)

Pb1—Se1	3.0553 (15)	Pb2—Se4^i^	2.900 (7)
Pb1—Se1^i^	3.0553 (15)	Pb2—Se6^i^	3.283 (8)
Pb1—Se2	2.9124 (17)	Pb2—Se6^vii^	3.283 (8)
Pb1—Se3^ii^	3.5754 (15)	Sb3—Se1	2.587 (2)
Pb1—Se3^iii^	3.5754 (15)	Sb3—Se2^vi^	2.9293 (15)
Pb1—Se4^ii^	3.4061 (16)	Sb3—Se2	2.9293 (15)
Pb1—Se5^iv^	3.1205 (17)	Sb3—Se3	2.8835 (14)
Pb1—Se5^v^	3.1205 (17)	Sb3—Se3^i^	2.8835 (14)
Sb1—Se1	2.95 (2)	Sb3—Se6^viii^	3.7015 (19)
Sb1—Se1^i^	2.95 (2)	Sb3—Se6^ix^	3.7015 (19)
Sb1—Se2	2.68 (3)	Pb3—Se1	3.02 (4)
Sb1—Se3^ii^	3.75 (2)	Pb3—Se2^vi^	3.05 (3)
Sb1—Se3^iii^	3.75 (2)	Pb3—Se2	3.05 (3)
Sb1—Se4^ii^	3.65 (2)	Pb3—Se3	2.86 (3)
Sb1—Se5^iv^	3.16 (3)	Pb3—Se3^i^	2.86 (3)
Sb1—Se5^v^	3.16 (3)	Pb3—Se6^viii^	3.35 (3)
Sb1—Cu1^v^	3.54 (3)	Pb3—Se6^ix^	3.35 (3)
Sb2—Se1^vi^	3.1563 (14)	Sb4—Se2^x^	3.166 (3)
Sb2—Se1	3.1563 (14)	Sb4—Se2^xi^	3.166 (3)
Sb2—Se3	2.6135 (19)	Sb4—Se5	2.595 (3)
Sb2—Se4	2.7090 (12)	Sb4—Se6	2.723 (2)
Sb2—Se4^i^	2.7090 (12)	Sb4—Se6^i^	2.723 (2)
Sb2—Se6^i^	3.6962 (17)	Sb4*a*—Se2^x^	3.30 (3)
Sb2—Se6^vii^	3.6962 (17)	Sb4*a*—Se2^xi^	2.77 (3)
Pb2—Se1^vi^	3.114 (7)	Sb4*a*—Se2^ii^	3.66 (2)
Pb2—Se1	3.114 (7)	Sb4*a*—Se5	2.633 (19)
Pb2—Se3	3.094 (10)	Sb4*a*—Se6	3.15 (3)
Pb2—Se4	2.900 (7)	Sb4*a*—Se6^i^	2.59 (3)

Symmetry codes: (i) 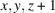 ; (ii) 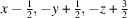 ; (iii) 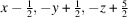 ; (iv) 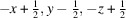 ; (v) 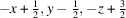 ; (vi) 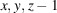 ; (vii) 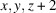 ; (viii) 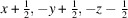 ; (ix) 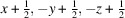 ; (x) 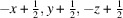 ; (xi) 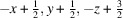 .

**Table 2:** Experimental details

Crystal data	
Chemical formula	CuPbSb_3_Se_6_
*M*_r_	1109.7
Crystal system, space group	Orthorhombic, *P* *n* *n* *m*
Temperature (K)	300
*a*, *b*, *c* (Å)	13.7217 (5), 20.5149 (8), 4.0716 (2)
*V* (Å^3^)	1146.15 (8)
*Z*	4
Radiation type	Mo *K*α
μ (mm^−1^)	42.44
Crystal size (mm)	0.08 × 0.06 × 0.04 × 0.03 (radius)

Data collection	
Diffractometer	Stoe Stadivari
Absorption correction	Multi-scan [absorption correction by scaling of reflection intensities followed by a spherical absorption correction (*LANA*; Koziskova *et al.*, 2016 ▸)]
*T*_min_, *T*_max_	0.428, 0.654
No. of measured, independent and observed [*I* > 3σ(*I*)] reflections	15049, 2534, 1472
*R*_int_	0.079
(sin θ/λ)_max_ (Å^−1^)	0.792

Refinement	
*R*[*F* > 3σ(*F*)], *wR*(*F*), *S*	0.042, 0.110, 1.40
No. of reflections	2534
No. of parameters	86
Δρ_max_, Δρ_min_ (e Å^−3^)	2.96, −2.68

Computer programs: *X-AREA Pilatus3_SV*, *Recipe*, *Integrate * and *LANA* (Stoe & Cie, 2021 ▸), *SHELXT* (Sheldrick, 2015 ▸), *JANA2006* (Petříček *et al.*, 2014 ▸), *DIAMOND* (Putz & Brandenburg, 2021 ▸) and *publCIF* (Westrip, 2010 ▸).

## supplementary crystallographic information

### Crystal data

**Table:**

CuPbSb_3_Se_6_	*F*(000) = 1872
*M**_r_* = 1109.7	*D*_x_ = 6.431 Mg m^−^^3^
Orthorhombic, *P**n**n**m*	Mo *K*α radiation, λ = 0.71073 Å
Hall symbol: -P -2xabc;-2yabc;-2z	Cell parameters from 17463 reflections
*a* = 13.7217 (5) Å	θ = 2.5–33.5°
*b* = 20.5149 (8) Å	µ = 42.44 mm^−^^1^
*c* = 4.0716 (2) Å	*T* = 300 K
*V* = 1146.15 (8) Å^3^	Fragment, black
*Z* = 4	0.08 × 0.06 × 0.04 × 0.03 (radius) mm

### Data collection

**Table:**

Stoe Stadivari diffractometer	2534 independent reflections
Radiation source: Axo_Mo	1472 reflections with *I* > 3σ(*I*)
Graded multilayer mirror monochromator	*R*_int_ = 0.079
Detector resolution: 13.33 pixels mm^-1^	θ_max_ = 34.3°, θ_min_ = 2.5°
rotation method, ω scans	*h* = −21→19
Absorption correction: multi-scan [absorption correction by scaling of reflection intensities followed by a spherical absorption correction (*LANA*; Koziskova *et al.*, 2016)]	*k* = −32→17
*T*_min_ = 0.428, *T*_max_ = 0.654	*l* = −6→4
15049 measured reflections	

### Refinement

**Table:**

Refinement on *F*^2^	0 restraints
*R*[*F* > 3σ(*F*)] = 0.042	24 constraints
*wR*(*F*) = 0.110	Weighting scheme based on measured s.u.'s *w* = 1/(σ^2^(*I*) + 0.0016*I*^2^)
*S* = 1.40	(Δ/σ)_max_ = 0.028
2534 reflections	Δρ_max_ = 2.96 e Å^−^^3^
86 parameters	Δρ_min_ = −2.68 e Å^−^^3^

### Fractional atomic coordinates and isotropic or equivalent isotropic displacement parameters (Å^2^)

**Table:**

	*x*	*y*	*z*	*U*_iso_*/*U*_eq_	Occ. (<1)
Pb1	0.22598 (7)	0.14687 (9)	1.5	0.0286 (2)	0.897 (4)
Sb1	0.2448 (17)	0.1462 (17)	1.5	0.0286 (2)	0.103 (4)
Sb2	0.40745 (12)	0.33892 (6)	0.5	0.0247 (3)	0.923 (3)
Pb2	0.3710 (7)	0.3449 (5)	0.5	0.0247 (3)	0.077 (3)
Sb3	0.49573 (14)	0.17280 (7)	1	0.0411 (4)	0.974 (5)
Pb3	0.527 (3)	0.1673 (19)	1	0.0411 (4)	0.026 (5)
Sb4	0.14104 (17)	0.47308 (17)	−0.5	0.0307 (11)	0.83 (3)
Sb4a	0.1341 (12)	0.4816 (13)	−0.402 (6)	0.0307 (11)	0.084 (15)
Se1	0.33027 (8)	0.23328 (6)	1	0.0199 (3)	
Se2	0.41682 (9)	0.08474 (6)	1.5	0.0228 (4)	
Se3	0.55995 (8)	0.26260 (6)	0.5	0.0209 (3)	
Se4	0.49064 (9)	0.40595 (6)	0	0.0214 (4)	
Se5	0.30698 (8)	0.53372 (6)	−0.5	0.0208 (3)	
Se6	0.19799 (9)	0.39357 (7)	−1	0.0297 (4)	
Cu1a	0.3449 (8)	0.4703 (6)	0	0.050 (3)	0.405 (17)
Cu1	0.3896 (5)	0.4990 (3)	0	0.0341 (14)	0.595 (17)

### Atomic displacement parameters (Å^2^)

**Table:**

	*U*^11^	*U*^22^	*U*^33^	*U*^12^	*U*^13^	*U*^23^
Pb1	0.0292 (5)	0.0306 (3)	0.0260 (3)	−0.0027 (5)	0	0
Sb1	0.0292 (5)	0.0306 (3)	0.0260 (3)	−0.0027 (5)	0	0
Sb2	0.0228 (6)	0.0241 (5)	0.0272 (5)	0.0007 (5)	0	0
Pb2	0.0228 (6)	0.0241 (5)	0.0272 (5)	0.0007 (5)	0	0
Sb3	0.0267 (6)	0.0335 (7)	0.0632 (8)	0.0089 (6)	0	0
Pb3	0.0267 (6)	0.0335 (7)	0.0632 (8)	0.0089 (6)	0	0
Sb4	0.0212 (5)	0.0344 (9)	0.037 (3)	−0.0042 (5)	0	0
Sb4a	0.0212 (5)	0.0344 (9)	0.037 (3)	−0.0042 (5)	0	0
Se1	0.0185 (5)	0.0209 (6)	0.0205 (6)	−0.0003 (5)	0	0
Se2	0.0183 (5)	0.0262 (7)	0.0241 (6)	−0.0016 (5)	0	0
Se3	0.0196 (5)	0.0208 (7)	0.0223 (6)	0.0003 (5)	0	0
Se4	0.0182 (5)	0.0220 (7)	0.0239 (6)	−0.0016 (4)	0	0
Se5	0.0197 (5)	0.0225 (7)	0.0200 (6)	−0.0012 (5)	0	0
Se6	0.0221 (6)	0.0257 (7)	0.0415 (8)	0.0009 (5)	0	0
Cu1a	0.044 (5)	0.057 (6)	0.048 (3)	0.025 (5)	0	0
Cu1	0.034 (3)	0.036 (3)	0.0331 (17)	0.009 (2)	0	0

### Geometric parameters (Å, º)

**Table:**

Pb1—Sb1	0.26 (2)	Pb3—Sb4a^viii^	3.41 (5)
Pb1—Se1	3.0553 (15)	Pb3—Sb4a^ix^	3.41 (5)
Pb1—Se1^i^	3.0553 (15)	Pb3—Se1	3.02 (4)
Pb1—Se2	2.9124 (17)	Pb3—Se2^vi^	3.05 (3)
Pb1—Se3^ii^	3.5754 (15)	Pb3—Se2	3.05 (3)
Pb1—Se3^iii^	3.5754 (15)	Pb3—Se3	2.86 (3)
Pb1—Se4^ii^	3.4061 (16)	Pb3—Se3^i^	2.86 (3)
Pb1—Se5^iv^	3.1205 (17)	Pb3—Se6^x^	3.35 (3)
Pb1—Se5^v^	3.1205 (17)	Pb3—Se6^viii^	3.35 (3)
Pb1—Cu1a^v^	3.751 (13)	Sb4—Sb4a^vi^	3.68 (3)
Pb1—Cu1^v^	3.423 (6)	Sb4—Sb4a	0.44 (3)
Sb1—Se1	2.95 (2)	Sb4—Sb4a^xi^	0.44 (3)
Sb1—Se1^i^	2.95 (2)	Sb4—Sb4a^xii^	3.68 (3)
Sb1—Se2	2.68 (3)	Sb4—Se2^xiii^	3.166 (3)
Sb1—Se3^ii^	3.75 (2)	Sb4—Se2^xiv^	3.166 (3)
Sb1—Se3^iii^	3.75 (2)	Sb4—Se5	2.595 (3)
Sb1—Se4^ii^	3.65 (2)	Sb4—Se6	2.723 (2)
Sb1—Se5^iv^	3.16 (3)	Sb4—Se6^i^	2.723 (2)
Sb1—Se5^v^	3.16 (3)	Sb4—Cu1a^vi^	3.461 (9)
Sb1—Cu1^v^	3.54 (3)	Sb4—Cu1a	3.461 (9)
Sb2—Pb2	0.515 (10)	Sb4a—Sb4a^xv^	3.76 (2)
Sb2—Se1^vi^	3.1563 (14)	Sb4a—Sb4a^xi^	0.79 (4)
Sb2—Se1	3.1563 (14)	Sb4a—Sb4a^xii^	3.28 (4)
Sb2—Se3	2.6135 (19)	Sb4a—Se2^xiii^	3.30 (3)
Sb2—Se4	2.7090 (12)	Sb4a—Se2^xiv^	2.77 (3)
Sb2—Se4^i^	2.7090 (12)	Sb4a—Se2^ii^	3.66 (2)
Sb2—Se6^i^	3.6962 (17)	Sb4a—Se5	2.633 (19)
Sb2—Se6^vii^	3.6962 (17)	Sb4a—Se6	3.15 (3)
Sb2—Cu1a	3.485 (10)	Sb4a—Se6^i^	2.59 (3)
Sb2—Cu1a^i^	3.485 (10)	Sb4a—Cu1a^vi^	3.79 (2)
Pb2—Se1^vi^	3.114 (7)	Sb4a—Cu1a	3.33 (2)
Pb2—Se1	3.114 (7)	Se1—Se3	3.7999 (14)
Pb2—Se3	3.094 (10)	Se1—Se3^i^	3.7999 (14)
Pb2—Se4	2.900 (7)	Se1—Se3^ii^	3.7101 (16)
Pb2—Se4^i^	2.900 (7)	Se1—Se6^vii^	3.7560 (19)
Pb2—Se6^i^	3.283 (8)	Se3—Se4	3.7009 (15)
Pb2—Se6^vii^	3.283 (8)	Se3—Se4^i^	3.7009 (15)
Pb2—Cu1a	3.300 (12)	Se3—Se6^x^	3.7219 (19)
Pb2—Cu1a^i^	3.300 (12)	Se4—Se5^xvi^	3.6590 (14)
Pb2—Cu1	3.770 (9)	Se4—Se5^xvii^	3.6590 (14)
Pb2—Cu1^i^	3.770 (9)	Se4—Cu1a	2.395 (12)
Sb3—Pb3	0.44 (4)	Se4—Cu1a^xvi^	3.397 (12)
Sb3—Sb4^viii^	3.596 (4)	Se4—Cu1	2.360 (6)
Sb3—Sb4a^viii^	3.71 (2)	Se4—Cu1^xvi^	2.550 (6)
Sb3—Sb4a^ix^	3.71 (2)	Se5—Cu1a^vi^	2.472 (7)
Sb3—Se1	2.587 (2)	Se5—Cu1a	2.472 (7)
Sb3—Se2^vi^	2.9293 (15)	Se5—Cu1^vi^	2.436 (4)
Sb3—Se2	2.9293 (15)	Se5—Cu1	2.436 (4)
Sb3—Se3	2.8835 (14)	Se6—Cu1a^vi^	2.558 (12)
Sb3—Se3^i^	2.8835 (14)	Se6—Cu1^vi^	3.405 (6)
Sb3—Se6^x^	3.7015 (19)	Cu1a—Cu1	0.851 (13)
Sb3—Se6^viii^	3.7015 (19)	Cu1a—Cu1^xvi^	3.696 (13)
Pb3—Sb4^viii^	3.28 (4)	Cu1—Cu1^xvi^	3.030 (9)
			
Se1—Pb1—Se1^i^	83.57 (5)	Se2^vi^—Sb3—Se6^x^	70.61 (4)
Se1—Pb1—Se2	80.37 (4)	Se2^vi^—Sb3—Se6^viii^	115.63 (6)
Se1—Pb1—Se3^ii^	67.52 (4)	Se2—Sb3—Se3	176.06 (8)
Se1—Pb1—Se3^iii^	112.11 (6)	Se2—Sb3—Se3^i^	90.93 (3)
Se1—Pb1—Se4^ii^	128.95 (3)	Se2—Sb3—Se6^x^	115.63 (6)
Se1—Pb1—Se5^iv^	93.71 (3)	Se2—Sb3—Se6^viii^	70.61 (4)
Se1—Pb1—Se5^v^	159.10 (5)	Se3—Sb3—Se3^i^	89.82 (5)
Se1^i^—Pb1—Se2	80.37 (4)	Se3—Sb3—Se6^x^	67.52 (4)
Se1^i^—Pb1—Se3^ii^	112.11 (6)	Se3—Sb3—Se6^viii^	113.22 (6)
Se1^i^—Pb1—Se3^iii^	67.52 (4)	Se3^i^—Sb3—Se6^x^	113.22 (6)
Se1^i^—Pb1—Se4^ii^	128.95 (3)	Se3^i^—Sb3—Se6^viii^	67.52 (4)
Se1^i^—Pb1—Se5^iv^	159.10 (5)	Se6^x^—Sb3—Se6^viii^	66.73 (4)
Se1^i^—Pb1—Se5^v^	93.71 (3)	Se1—Pb3—Se2^vi^	78.8 (8)
Se2—Pb1—Se3^ii^	143.15 (2)	Se1—Pb3—Se2	78.8 (8)
Se2—Pb1—Se3^iii^	143.15 (2)	Se1—Pb3—Se3	80.5 (8)
Se2—Pb1—Se4^ii^	135.50 (7)	Se1—Pb3—Se3^i^	80.5 (8)
Se2—Pb1—Se5^iv^	78.74 (5)	Se1—Pb3—Se6^x^	142.5 (4)
Se2—Pb1—Se5^v^	78.74 (5)	Se1—Pb3—Se6^viii^	142.5 (4)
Se3^ii^—Pb1—Se3^iii^	69.42 (3)	Se2^vi^—Pb3—Se2	83.8 (9)
Se3^ii^—Pb1—Se4^ii^	63.97 (3)	Se2^vi^—Pb3—Se3	89.0 (2)
Se3^ii^—Pb1—Se5^iv^	85.56 (3)	Se2^vi^—Pb3—Se3^i^	159.1 (13)
Se3^ii^—Pb1—Se5^v^	131.72 (4)	Se2^vi^—Pb3—Se6^x^	74.6 (5)
Se3^iii^—Pb1—Se4^ii^	63.97 (3)	Se2^vi^—Pb3—Se6^viii^	123.1 (12)
Se3^iii^—Pb1—Se5^iv^	131.72 (4)	Se2—Pb3—Se3	159.1 (13)
Se3^iii^—Pb1—Se5^v^	85.56 (3)	Se2—Pb3—Se3^i^	89.0 (2)
Se4^ii^—Pb1—Se5^iv^	68.04 (4)	Se2—Pb3—Se6^x^	123.1 (12)
Se4^ii^—Pb1—Se5^v^	68.04 (4)	Se2—Pb3—Se6^viii^	74.6 (5)
Se5^iv^—Pb1—Se5^v^	81.45 (5)	Se3—Pb3—Se3^i^	90.8 (11)
Se1—Sb1—Se1^i^	87.2 (8)	Se3—Pb3—Se6^x^	73.2 (5)
Se1—Sb1—Se2	86.2 (6)	Se3—Pb3—Se6^viii^	125.2 (12)
Se1—Sb1—Se3^ii^	66.0 (4)	Se3^i^—Pb3—Se6^x^	125.2 (12)
Se1—Sb1—Se3^iii^	110.0 (9)	Se3^i^—Pb3—Se6^viii^	73.2 (5)
Se1—Sb1—Se4^ii^	123.9 (6)	Se6^x^—Pb3—Se6^viii^	74.9 (7)
Se1—Sb1—Se5^iv^	94.99 (14)	Se2^xiii^—Sb4—Se2^xiv^	80.04 (8)
Se1—Sb1—Se5^v^	167.5 (10)	Se2^xiii^—Sb4—Se5	82.71 (8)
Se1^i^—Sb1—Se2	86.2 (6)	Se2^xiii^—Sb4—Se6	91.41 (3)
Se1^i^—Sb1—Se3^ii^	110.0 (9)	Se2^xiii^—Sb4—Se6^i^	170.44 (11)
Se1^i^—Sb1—Se3^iii^	66.0 (4)	Se2^xiv^—Sb4—Se5	82.71 (8)
Se1^i^—Sb1—Se4^ii^	123.9 (6)	Se2^xiv^—Sb4—Se6	170.44 (11)
Se1^i^—Sb1—Se5^iv^	167.5 (10)	Se2^xiv^—Sb4—Se6^i^	91.41 (3)
Se1^i^—Sb1—Se5^v^	94.99 (14)	Se5—Sb4—Se6	92.03 (7)
Se2—Sb1—Se3^ii^	146.2 (3)	Se5—Sb4—Se6^i^	92.03 (7)
Se2—Sb1—Se3^iii^	146.2 (3)	Se6—Sb4—Se6^i^	96.76 (11)
Se2—Sb1—Se4^ii^	134.8 (12)	Se2^xiii^—Sb4a—Se2^xiv^	83.9 (7)
Se2—Sb1—Se5^iv^	81.6 (7)	Se2^xiii^—Sb4a—Se2^ii^	113.3 (5)
Se2—Sb1—Se5^v^	81.6 (7)	Se2^xiii^—Sb4a—Se5	79.6 (6)
Se3^ii^—Sb1—Se3^iii^	65.7 (5)	Se2^xiii^—Sb4a—Se6	81.8 (6)
Se3^ii^—Sb1—Se4^ii^	60.0 (3)	Se2^xiii^—Sb4a—Se6^i^	170.0 (9)
Se3^ii^—Sb1—Se5^iv^	82.1 (3)	Se2^xiv^—Sb4a—Se2^ii^	79.3 (5)
Se3^ii^—Sb1—Se5^v^	124.2 (7)	Se2^xiv^—Sb4a—Se5	90.4 (8)
Se3^iii^—Sb1—Se4^ii^	60.0 (3)	Se2^xiv^—Sb4a—Se6	164.8 (10)
Se3^iii^—Sb1—Se5^iv^	124.2 (7)	Se2^xiv^—Sb4a—Se6^i^	104.1 (9)
Se3^iii^—Sb1—Se5^v^	82.1 (3)	Se2^ii^—Sb4a—Se5	162.1 (9)
Se4^ii^—Sb1—Se5^iv^	64.6 (5)	Se2^ii^—Sb4a—Se6	111.0 (7)
Se4^ii^—Sb1—Se5^v^	64.6 (5)	Se2^ii^—Sb4a—Se6^i^	74.6 (6)
Se5^iv^—Sb1—Se5^v^	80.3 (8)	Se5—Sb4a—Se6	82.3 (6)
Se1^vi^—Sb2—Se1	80.33 (4)	Se5—Sb4a—Se6^i^	94.2 (6)
Se1^vi^—Sb2—Se3	81.80 (4)	Se6—Sb4a—Se6^i^	89.7 (7)
Se1^vi^—Sb2—Se4	90.30 (3)	Se3—Se1—Se3^i^	64.79 (2)
Se1^vi^—Sb2—Se4^i^	167.07 (5)	Se3—Se1—Se3^ii^	145.65 (2)
Se1^vi^—Sb2—Se6^i^	65.93 (4)	Se3—Se1—Se6^vii^	105.20 (4)
Se1^vi^—Sb2—Se6^vii^	107.62 (5)	Se3^i^—Se1—Se3^ii^	145.65 (2)
Se1—Sb2—Se3	81.80 (4)	Se3^i^—Se1—Se6^vii^	105.20 (4)
Se1—Sb2—Se4	167.07 (5)	Se3^ii^—Se1—Se6^vii^	59.80 (3)
Se1—Sb2—Se4^i^	90.30 (3)	Se1^vi^—Se3—Se1	64.79 (2)
Se1—Sb2—Se6^i^	107.62 (5)	Se1^vi^—Se3—Se1^xviii^	146.387 (17)
Se1—Sb2—Se6^vii^	65.93 (4)	Se1^vi^—Se3—Se4	67.54 (3)
Se3—Sb2—Se4	88.09 (5)	Se1^vi^—Se3—Se4^i^	101.97 (4)
Se3—Sb2—Se4^i^	88.09 (5)	Se1^vi^—Se3—Se6^x^	106.61 (4)
Se3—Sb2—Se6^i^	143.55 (3)	Se1—Se3—Se1^xviii^	146.387 (17)
Se3—Sb2—Se6^vii^	143.55 (3)	Se1—Se3—Se4	101.97 (4)
Se4—Sb2—Se4^i^	97.44 (5)	Se1—Se3—Se4^i^	67.54 (3)
Se4—Sb2—Se6^i^	76.10 (3)	Se1—Se3—Se6^x^	106.61 (4)
Se4—Sb2—Se6^vii^	125.99 (5)	Se1^xviii^—Se3—Se4	103.82 (4)
Se4^i^—Sb2—Se6^i^	125.99 (5)	Se1^xviii^—Se3—Se4^i^	103.82 (4)
Se4^i^—Sb2—Se6^vii^	76.10 (3)	Se1^xviii^—Se3—Se6^x^	60.71 (3)
Se6^i^—Sb2—Se6^vii^	66.84 (3)	Se4—Se3—Se4^i^	66.75 (3)
Se1^vi^—Pb2—Se1	81.7 (2)	Se4—Se3—Se6^x^	144.56 (2)
Se1^vi^—Pb2—Se3	75.5 (2)	Se4^i^—Se3—Se6^x^	144.56 (2)
Se1^vi^—Pb2—Se4	87.72 (9)	Se3^vi^—Se4—Se3	66.75 (3)
Se1^vi^—Pb2—Se4^i^	151.4 (4)	Se3^vi^—Se4—Se5^xvi^	76.57 (3)
Se1^vi^—Pb2—Se6^i^	71.86 (13)	Se3^vi^—Se4—Se5^xvii^	112.32 (4)
Se1^vi^—Pb2—Se6^vii^	120.0 (3)	Se3—Se4—Se5^xvi^	112.32 (4)
Se1—Pb2—Se3	75.5 (2)	Se3—Se4—Se5^xvii^	76.57 (3)
Se1—Pb2—Se4	151.4 (4)	Se5^xvi^—Se4—Se5^xvii^	67.61 (3)
Se1—Pb2—Se4^i^	87.72 (9)	Se4^xix^—Se5—Se4^xvi^	67.61 (3)
Se1—Pb2—Se6^i^	120.0 (3)	Se1^xx^—Se6—Se3^xxi^	59.49 (3)
Se1—Pb2—Se6^vii^	71.86 (13)	Se4—Cu1a—Se4^xvi^	81.8 (3)
Se3—Pb2—Se4	76.2 (2)	Se4—Cu1a—Se5	117.8 (3)
Se3—Pb2—Se4^i^	76.2 (2)	Se4—Cu1a—Se5^i^	117.8 (3)
Se3—Pb2—Se6^i^	140.54 (13)	Se4—Cu1a—Se6^i^	108.6 (5)
Se3—Pb2—Se6^vii^	140.54 (13)	Se4^xvi^—Cu1a—Se5	75.3 (3)
Se4—Pb2—Se4^i^	89.2 (3)	Se4^xvi^—Cu1a—Se5^i^	75.3 (3)
Se4—Pb2—Se6^i^	80.94 (12)	Se4^xvi^—Cu1a—Se6^i^	169.6 (5)
Se4—Pb2—Se6^vii^	135.5 (3)	Se5—Cu1a—Se5^i^	110.9 (5)
Se4^i^—Pb2—Se6^i^	135.5 (3)	Se5—Cu1a—Se6^i^	99.1 (3)
Se4^i^—Pb2—Se6^vii^	80.94 (12)	Se5^i^—Cu1a—Se6^i^	99.1 (3)
Se6^i^—Pb2—Se6^vii^	76.7 (2)	Se4—Cu1—Se4^xvi^	103.9 (2)
Se1—Sb3—Se2^vi^	88.36 (6)	Se4—Cu1—Se5	120.65 (13)
Se1—Sb3—Se2	88.36 (6)	Se4—Cu1—Se5^i^	120.65 (13)
Se1—Sb3—Se3	87.81 (5)	Se4—Cu1—Se6^i^	86.53 (17)
Se1—Sb3—Se3^i^	87.81 (5)	Se4^xvi^—Cu1—Se5	94.39 (16)
Se1—Sb3—Se6^x^	146.549 (19)	Se4^xvi^—Cu1—Se5^i^	94.39 (16)
Se1—Sb3—Se6^viii^	146.549 (19)	Se4^xvi^—Cu1—Se6^i^	169.6 (2)
Se2^vi^—Sb3—Se2	88.05 (5)	Se5—Cu1—Se5^i^	113.3 (2)
Se2^vi^—Sb3—Se3	90.93 (3)	Se5—Cu1—Se6^i^	79.99 (16)
Se2^vi^—Sb3—Se3^i^	176.06 (8)	Se5^i^—Cu1—Se6^i^	79.99 (16)

Symmetry codes: (i) *x*, *y*, *z*+1; (ii) *x*−1/2, −*y*+1/2, −*z*+3/2; (iii) *x*−1/2, −*y*+1/2, −*z*+5/2; (iv) −*x*+1/2, *y*−1/2, −*z*+1/2; (v) −*x*+1/2, *y*−1/2, −*z*+3/2; (vi) *x*, *y*, *z*−1; (vii) *x*, *y*, *z*+2; (viii) *x*+1/2, −*y*+1/2, −*z*+1/2; (ix) *x*+1/2, −*y*+1/2, *z*+3/2; (x) *x*+1/2, −*y*+1/2, −*z*−1/2; (xi) *x*, *y*, −*z*−1; (xii) *x*, *y*, −*z*; (xiii) −*x*+1/2, *y*+1/2, −*z*+1/2; (xiv) −*x*+1/2, *y*+1/2, −*z*+3/2; (xv) −*x*, −*y*+1, *z*; (xvi) −*x*+1, −*y*+1, *z*; (xvii) −*x*+1, −*y*+1, *z*+1; (xviii) *x*+1/2, −*y*+1/2, −*z*+3/2; (xix) −*x*+1, −*y*+1, *z*−1; (xx) *x*, *y*, *z*−2; (xxi) *x*−1/2, −*y*+1/2, −*z*−1/2.
